# Association of tumor necrosis factor alpha gene polymorphism G-308A with pseudoexfoliative glaucoma in the Pakistani population

**Published:** 2009-12-22

**Authors:** Muhammad Imran Khan, Shazia Micheal, Noreen Rana, Farah Akhtar, Anneke I. den Hollander, Asifa Ahmed, Raheel Qamar

**Affiliations:** 1 Department of Biosciences, COMSATS Institute of Information Technology, Islamabad, Pakistan; 2 Department of Human Genetics, Radboud University Nijmegen Medical Centre, Nijmegen, The Netherlands; 3 Shifa College of Medicine, Islamabad, Pakistan; 4 Al-Shifa Trust Eye Hospital Rawalpindi, Pakistan; 5 Department of Ophthalmology, Radboud University Nijmegen Medical Centre, Nijmegen, The Netherlands

## Abstract

**Purpose:**

The purpose of the present study was to determine the role of the tumor necrosis factor alpha (*TNF-α*) gene polymorphism G-308A and total serum immunoglobulin E (TsIgE) levels in the onset of pseudoexfoliation glaucoma (PEXG) in Pakistani patients.

**Methods:**

The *TNF-α* polymorphism G-308A was analyzed in 122 patients with PEXG and 126 healthy unrelated controls by using polymerase chain reaction (PCR) and restriction fragment length polymorphism (RFLP). TsIgE levels were determined by solid-phase enzyme-linked immunosorbent assay (ELISA).

**Results:**

The AA and GA genotypes were strongly associated with PEXG (p<0.001), with an odds ratio (OR) of 0.07 (95% confidence interval [CI]=0.02-0.27) and 0.24 (95% CI=0.12-0.51), respectively, while the GG genotype was found at a higher frequency in controls as compared to patients (p<0.001) OR=8.95 (95% CI=4.55–17.81). No significant difference was found in TsIgE levels of both patients and controls (p=0.86).

**Conclusion:**

The present study concludes that the *TNF-α* polymorphism G-308A is strongly associated with PEXG. To our knowledge this is the first study in southeast Asia which demonstrates a strong association of a *TNF-α* polymorphism with PEXG.

## Introduction

Pseudoexfoliation syndrome (PEX) was first described by Lindberg in 1917 and involves excessive production and progressive accumulation of fibrillar extracellular material in various tissues [1,2]. Although the syndrome is recognized to be a systemic disorder, it is the most common identifiable cause of glaucoma that has now been termed pseudoexfoliation glaucoma (PEXG) [2–4].

PEX syndrome occurs worldwide but its prevalence varies among different age groups, races, and geographical locations. The highest prevalence of 20–70% has been reported in elderly people (70–80 years) and it is estimated that PEXG affects 10– 20% of all individuals above 60 years of age. Moreover, prevalence varies in different countries with the highest rate of about 46.9% in Greece [5]. Comprehensive data on the prevalence of PEXG in Pakistan is not available. However, a recently conducted hospital-based study did reveal a high rate of PEXG (6.45%) among patients with ocular ailments [6]. The mechanism underlying the development of PEX syndrome and its subsequent progression to PEXG is still unclear [4,7]. Studies have focused on immunological changes during glaucomatous pathogenesis and possible preventive therapies based along those lines have been proposed. The major targets of interest are cytokines, as recent advances reveal that they play an important role in the pathogenesis of glaucoma and may regulate retinal ganglion cell (RGC) survival or death [8].

Tumor necrosis factor alpha (TNF-α), a proinflammatory cytokine, is upregulated in several neurodegenerative disorders including multiple sclerosis, Parkinson’s disease, Alzheimer’s disease [9,10] and in optic nerve microglia and astrocytes in glaucoma patients [11,12]. It has been observed that both mRNA and protein levels of *TNF-α* or TNF-α receptor-1 (*TNF-R1*) are raised in the retina of glaucomatous eyes as compared to normal eyes, and therefore it was suggested that cell death mediated by TNF-α is a contributing factor in the neurodegeneration in glaucoma [13]. Using animal models, Nakazawa et al. [14] showed that ocular hypertension or increased intraocular pressure induces *TNF-α* upregulation in the retina, which in turn leads to RGC degeneration. Moreover, it has been observed that anti-TNF-α antibodies can prevent death of RGCs by reducing ocular hypertension, suggesting that reducing the expression of TNF-α would be beneficial in treating glaucoma [14].

Several polymorphisms have been identified in the upstream regulatory region of *TNF-α*. Among these is a common biallelic G (TNF1 allele) transition to A (TNF2 allele) 308 (rs1800629) nucleotide upstream from the transcription initiation site in the *TNF-α* promoter. This transition is considered to be an important enhancer of transcriptional activation associated with elevated levels of TNF-α [15], which have been shown to be involved in increased susceptibility to different eye diseases including diabetic retinopathy and glaucoma [16–18]. Association studies of the G-308A polymorphism in different populations have shown contradictory results [19,20]. Therefore, additional comprehensive studies involving different populations are warranted to better understand the role of *TNF-α* polymorphisms in different diseases. The aim of the present study was to study the association of the *TNF-α* polymorphism G-308A with PEXG in the Pakistani population.

## Methods

This was a multicenter case-control study that included 122 PEXG patients and 126 controls. The study was approved by the Departmental Ethical Committee of COMSATS Institute of Informational Technology and the relevant hospital ethics committees, and conformed to the principles of the Declaration of Helsinki. PEXG patients were recruited from the outpatient departments of two major eye clinics including Al-Shifa Eye Trust Hospital, Rawalpindi, and Christian Eye Hospital, Taxila. The healthy control individuals did not have any exfoliation material in the eye or any other evidence of glaucoma, and had normal visual fields.

All the PEXG patients underwent a complete ophthalmic examination including tonometric assessment of intraocular pressure (IOP), measurement of cup to disk ratio (CDR), and slit lamp biomicroscopy. Patients were evaluated by slit lamp biomicroscopy for the presence of exfoliative material along the papillary border and on the iris without dilating the eye. After pupil dilation the patients were also analyzed for the presence of white material deposits on the anterior lens surface. Gonioscopy was performed in all patients with a high magnification lens for determination of the angle. Approximately 4–6 ml of venous blood was obtained in vacutainers (product no 364606, Becton Dickinson, Franklin Lakes, NJ) containing acid citrate dextrose (ACD) as anticoagulant, after obtaining informed written consent from the subjects. For the ELISA assay, serum was obtained from clotted blood collected in Z-serum sep clot-activator vacutainer tubes (Greiner Bio-One, Munich, Germany) by centrifuging the vacutainers at 3,000 rpm for 10 min.

Genomic DNA was isolated by a standard phenol–chloroform extraction method. A 212 bp DNA sequence corresponding to the promoter region of *TNF-α* was amplified using the forward primer 5′-AGG CAA TAG GTT TTG AGG G***C***C AT-3′ and the reverse primer 5′-GTA GTG GGC CCT GCA CCT TCT-3′ [21]. The forward primer contains a C (bold italic) which is not present in the genomic sequence, and was introduced to create the NcoI enzyme recognition site (i.e. CCATGG) in the normal sequence. A transition from G to A abolishes the NcoI site in the mutant allele. 

PCR was performed in a final volume of 25 µl containing 40–50 ng of genomic DNA, 0.2 mM deoxynucleotide triphosphates (dNTPs), 1.5 mM MgCl_2,_ 0.2 µM each of reverse and forward primer, 1× Taq buffer, and 1 U of Taq polymerase. Polymerase chain reaction (PCR) was performed in a thermal cycler by initial denaturation of the genomic DNA at 95 °C for 7 min, followed by 35 cycles of denaturation at 95 °C for 30 s, primer annealing at 60 °C for 30 s, and chain extension at 72 °C for 45 s. A final extension step was performed at 72 °C for 10 min.

The PCR product (12 µl) was subjected to restriction enzyme digestion at 37 °C overnight with 10 U of NcoI restriction enzyme according to the manufacturer’s instructions (Fermentas, Burlington, Ontario). The resulting digested products were resolved on 4% agarose gels. PCR products carrying the variant A allele remained uncut (212 bp), while PCR products carrying the wild-type G allele were digested into two fragments of 192 bp and 20 bp.

TsIgE levels of patients and controls were determined using the immunoglobulin (IgE) enzyme immunoassay quantitative test kit BC-1035 (BioCheck Inc., Foster City, CA). TsIgE levels of all the patients and healthy control individuals were measured in duplicate.

A contingency table analysis (χ2 test) was performed to determine the level of statistical association between genotype and allele frequencies in patients and controls using StatCalc from Epi Info, Version 6. TsIgE levels were expressed as arithmetic or geometric means in IU/ml. To obtain a Gaussian distribution, TsIgE levels were normalized by logarithmic transformation (Log_10_). Statistical computations were also performed using Statistical Package for the Social Sciences (SPSS version 16). A p-value <0.05 was considered statistically significant.

## Results

A total of 248 individuals (122 PEXG patients and 126 unrelated healthy control individuals) were included in the present study. The PEXG patients had a mean age of 45.3±12.7 years and included 67% males and 33% females, whereas the controls had a mean age of 44.2±12.3 years with 64% males and 36% females (Table 1).

**Table 1:** *TNF-α* G −308A genotype and allele frequency distribution between patients and controls.

** **	**Controls (126)**	**PEXG (122)**	**OR (95% CI)**	**p (χ^2^)**
Mean age (years)	44.2±12.3	45.3±12.7		
**Gender distribution**				
Males	81 (64.3%)	82 (67.2%)	0.88 (0.50–1.54)	0.6 (0.24)
Female	45 (35.7%)	40 (32.8%)	** **	** **
**Genotype and allele frequency distribution**				
**Genotypes (Males+Females)**				
GG	110 (87.3%)	53 (43.4%)	8.95 (4.55–17.81)	<0.001 (54.97)
GA	13 (10.3%)	39 (32.0%)	0.24 (0.12–0.51)	** **
AA	3 (2.4%)	30 (24.6%)	0.07 (0.02–0.27)	** **
**Alleles (Males+Females)**				
G	233 (92.5%)	145 (59.4%)	8.37 (4.78–14.81)	<0.001 (74.61)
A	19 (7.5%)	99 (40.6%)	** **	** **
**Genotypes (Males)**				
GG	69 (85.2%)	36 (43.9%)	7.35 (3.27–16.79)	<0.001 (33.30)
GA	11 (13.6%)	27 (32.9%)	0.32 (0.14–0.75)	** **
AA	1 (1.2%)	19 (23.2%)	0.04 (0.001–0.31)	** **
**Alleles (Males)**				
G	149 (92.0%)	99(60.4%)	7.53 (3.79–15.19)	<0.001 (44.74)
A	13 (8.0%)	65(39.6%)	** **	** **
**Genotypes (Females)**				
GG	41 (91.1%)	17 (42.5%)	13.87 (3.74–56.25)	<0.001 (23.09)
GA	2 (4.4%)	12 (30.0%)	0.11 (0.02–0.58)	** **
AA	2 (4.4%)	11 (27.5%)	0.12 (0.02–0.66)	** **
**Alleles (Females)**				
G	84 (93.3%)	46 (57.5%)	10.35 (3.78–29.81)	<0.001 (30.22)
A	6 (6.7%)	34 (42.5%)	** **	** **

Values in parentheses represent percentage.

The GG wild-type genotype was found at a frequency of 87% in the controls and 43% in the PEXG patients with an odds ratio (OR) of 8.95 (95% confidence interval [CI]=4.55–17.81; Table 1), whereas the number of heterozygous and homozygous variant individuals was higher in patients as compared to controls (p<0.001, χ^2^ 54.97). Of the PEXG patients, 25% contained the AA variant genotype while in the controls this genotype was present at only 2%, with OR=0.07 (95% CI=0.02–0.27). The GA heterozygous genotype was present in 32% of the PEXG patients, whereas only 10% of the controls were heterozygous (OR=0.24 [95% CI=0.12–0.51]). The distribution of allele frequencies (Table 1) of the patients and controls showed that the A allele was more prevalent in the PEXG patients (41%) as compared to controls (8%) and was strongly associated with the disease condition, OR=8.37 (95% CI=4.78–14.81; p<0.001, χ^2^ 74.6).

The genotype distribution data were also stratified with respect to gender to determine any gender bias (Table 1). Even while considering the data of males and females separately, the GA and AA genotypes remained significantly associated with PEXG, (p<0.001, χ^2^=33.30 and p<0.001, χ^2^=23.09, respectively). In addition, the variant A allele was found to be more prevalent in the PEXG male patients as well as female patients when compared to the appropriate controls (p<0.001; χ^2^=44.74, χ^2^=30.22, respectively). The frequency of the A allele was found to be nearly the same in both sexes (40% and 43%, respectively; Table 1).

TsIgE values were found to follow a log normal distribution in both the cohorts: controls and PEXG patients (Table 2). No significant difference was found between the TsIgE levels of PEXG patients and controls (Table 2A). In addition, no significant difference was found when the Log_10_ TsIgE values of the different genotypes of the G-308A polymorphism (GG, GA, and AA) of patients and controls were compared (Table 2B).

**Table 2:** Comparison of mean TsIgE levels in controls and patients, and among different genotypes of *TNF-α* polymorphism G −308A.

**A. Comparison of mean log of TsIgE levels in controls and patients**			
TsIgE in IU/ml	Controls (83) Mean±SD	PEXG (40) Mean±SD	p–value
** **	1.96±0.52	1.94±0.63	0.86
**B. Comparison of log of TsIgE levels in genotypes of controls and patients**			
Genotypes	Controls (83) Mean±SD	Patients (40) Mean±SD	p-value
GG	1.98±0.53	2.02±0.62	0.84
GA	1.76±0.47	2.06±0.64	0.2
AA	2.02±0.49	1.76±0.63	0.49

## Discussion

During the last few years, several immunological components have been shown to be involved in the pathogenesis of various types of glaucoma. It has been observed that these components are not usually the primary causative agents but are involved in the progression of the disease [21,22]. In addition, it has been reported that components of the immune system involved in the pathogenesis of glaucoma are also involved in neurodegeneration following brain injury. In such cases inflammation occurs in response to glutamate, reactive oxygen species (ROS), nitric oxide (NO), and cytokines including tumor necrosis factor (TNF)-α, which are released from activated microglia or macrophages [23]. As glaucoma is a disease of old age and involves optic nerve neuropathy, it has been proposed that both genetic as well as epigenetic factors are involved in the progression of the disease. Such factors cause a decrease in the cellular viability and self-renewal capacity, which results in the generation of dysfunctional microglia. Such age-related attrition may contribute to the development of neurodegenerative diseases by diminishing glial neurosupportive functions. Secondary degeneration by the immune components leads to the neurodegenerative injury in glaucoma [24].

TNF-α is considered to be a neuroprotective component of the immune system, because it activates the ubiquitous transcription factor NF-κB through binding to the high affinity TNF receptor (TNF-R2), which in turn mediates the expression of a wide range of genes essential for neuronal survival. Contrary to its neuroprotective role, TNF-α can also serve as a neurodegenerative factor when it binds to the low affinity death receptor TNF-R1 and induces the mitochondria-mediated apoptotic pathway [25–29]. Thus a delicate balance between the two pathways determines the survival of the cell, and any shift in equilibrium might have deleterious effects. An increased expression of TNF-α can shift the balance toward TNF-R1 signaling, as seen in glaucoma, and thus promote retinal ganglion cell death. Based on the latter observation, an antiglaucoma drug, GLC756, has been developed that inhibits TNF-α release from activated rat mast cells and thus promotes cell survival. This can be attributed to the potential neuroprotective role of the compound (GLC756), which can therefore serve an important role in the management of glaucoma [30,31].

In the present study we found a strong association of GA and AA genotypes with PEXG, suggesting a role of the A allele in the pathogenesis of the disease. However, our results are in contradiction with previous studies, which have shown no significant association of the *TNF-α* polymorphism G-308A with PEXG in both Turkish and Caucasian populations [32,33]. Agarwal et al. [34] reported that the G to A transition at position −308 results in a six- to sevenfold increase in transcription of *TNF-α* as compared to normal basal level transcription, as a result of which increased levels of TNF were observed. Recently, a significant association has been observed for the G-308A polymorphism between PEXG patients and controls in the Iranian population (p*<*0.005) [35], which is in agreement with our results. This similarity with the Iranian population has also been observed for the association of the ABO blood groups with different types of glaucoma. Blood group B was strongly associated with different types of glaucoma [36], which is in agreement with the study of Zaree et al. [37] in the Iranian population. Genetic relatedness was also demonstrated previously in studies of the Y chromosomal markers, which have shown that the Pakistani populations share a significant similarity in their genetic makeup with different Iranian ethnic groups [38].

Our results are in accordance with the findings of Razenghinejad et al. [35], and are interesting for several reasons. In our study the statistically significant differences between patients and controls are more pronounced as compared to the Iranian population, (<0.001 versus p=0.002). We attribute this difference to the fact that we not only observed a very high frequency of the heterozygous GA allele compared to the Iranian study (32% versus 17%), but also found the derived AA allele at a much higher frequency (24.6% versus 1.7%). Razenghinejad et al. [35] also noted that when the data was stratified according to gender, the genotype frequency became statistically insignificant for females, an observation that they attributed to the unequal gender distribution in the study subjects. As opposed to the Iranian study, we find that both the genotype and allele frequencies remain significantly associated with the disease even after gender-wise stratification (Table 1). A likely explanation is that gender matching was better in our study than in the Iranian study, thus emphasizing the importance of careful design of genetic association studies.

A strong association of this polymorphism has also been observed in the Chinese population, but that study was performed in patients with primary open-angle glaucoma (POAG) and with a very small sample size [20]. Although several investigators were unable to show a significant difference in genotype distribution and allele frequency between patients and control groups, some investigators have successfully demonstrated the functionality of this polymorphism in the reporter gene assays with a significant upregulation of up to fivefold in the constructs of TNF1 and TNF2 alleles [39]. This suggests that transcriptional regulation of *TNF-α* is essential to circumvent the deleterious effects of overexpression by transcriptional upregulation. Thus excessive production of TNF associated with the G-308A polymorphism may have an important role in the development of TNF2-associated autoimmune diseases, and may act as a genetic susceptibility factor driven by a high TNF-α expression, which would subsequently lead to immune responses causing the onset of different diseases. A recent study by Sawada et al. [40] also demonstrates that TNF-α levels were significantly elevated in the aqueous humor of PEXG patients as compared to controls and other glaucoma subtypes, including POAG and normal tension glaucoma. We believe that this is in line with our conjecture that TNF-α is significantly involved in the pathogenesis of the disease in the Pakistani population.

It is worth mentioning here that the greater number of carriers with a GA genotype in the PEXG patients also points toward the possibility of a dominant mode of inheritance of the genetic defects responsible for PEXG. However, due to the presence of such carriers in normal individuals, although at much lower frequency, it is likely that environmental influences and other genetic factors contribute to the development of the disease.

On the basis of different evidence it has been suggested that autoimmune damage to the optic nerve may occur directly by autoantibodies or indirectly by a “mimicked” autoimmune response to a sensitizing antigen, which in turn injures retinal ganglion cells [41]. In a recent study by Joachim et al. [22], IgG antibody levels were found to be significantly higher in the aqueous humor of patients with POAG and PEXG as compared to controls, due to which it was concluded that multiple factors contribute to the immune status of the eye [22]. Therefore we also investigated the role of TsIgE levels in glaucoma and its association with the G-308A polymorphism, as previously in a different disease it has been shown that IgE levels were associated with this single nucleotide polymorphism [42]. However, we did not find any association of IgE levels with PEXG in our study population, which could be because there is no significant involvement of TsIgE levels in the manifestation and onset of the disease. This warrants further investigation simultaneously in the serum and aqueous humor of patients, as previously pointed out by Joachim et al. [22].

In conclusion, we find the A allele of the *TNF-α* polymorphism G-308A to be strongly associated with the pathogenesis of PEXG, and thus propose that the GA and AA genotypes of the *TNF-α* regulatory region can be considered genetic markers for the stratification of PEXG patients in Pakistan. Mutations in *TNF-α* might be involved in causing different neurodegenerative disorders, therefore it is important to consider it as a novel therapeutic target for the treatment of neurodegenerative diseases like glaucoma. We expect that our study will prove to be a helpful tool in the treatment and management of PEXG by opening up new horizons in the field of target discovery to develop novel therapeutics.
